# Aspirin Mitigated Tumor Growth in Obese Mice Involving Metabolic Inhibition

**DOI:** 10.3390/cells9030569

**Published:** 2020-02-28

**Authors:** Jiaan-Der Wang, Wen-Ying Chen, Jian-Ri Li, Shih-Yi Lin, Ya-Yu Wang, Chih-Cheng Wu, Su-Lan Liao, Chiao-Chen Ko, Chun-Jung Chen

**Affiliations:** 1 Children’s Medical Center, Taichung Veterans General Hospital, Taichung City 407, Taiwan; wangjiaander@gmail.com; 2 Department of Industrial Engineering and Enterprise Information, Tunghai University, Taichung City 407, Taiwan; 3 Department of Veterinary Medicine, National Chung Hsing University, Taichung City 402, Taiwan; wychen@dragon.nchu.edu.tw (W.-Y.C.); angela51934@hotmail.com (C.-C.K.); 4 Division of Urology, Taichung Veterans General Hospital, Taichung City 407, Taiwan; fisherfishli@yahoo.com.tw; 5 Center for Geriatrics and Gerontology, Taichung Veterans General Hospital, Taichung City 407, Taiwan; sylin@vghtc.gov.tw; 6 Institute of Clinical Medicine, National Yang Ming University, Taipei City 112, Taiwan; yywang@vghtc.gov.tw; 7 Department of Family Medicine, Taichung Veterans General Hospital, Taichung City 407, Taiwan; 8 Department of Anesthesiology, Taichung Veterans General Hospital, Taichung City 407, Taiwan; chihcheng.wu@gmail.com; 9 Department of Financial Engineering, Providence University, Taichung City 433, Taiwan; 10 Department of Data Science and Big Data Analytics, Providence University, Taichung City 433, Taiwan; 11 Department of Medical Research, Taichung Veterans General Hospital, Taichung City 407, Taiwan; slliao@vghtc.gov.tw; 12 Department of Medical Laboratory Science and Biotechnology, China Medical University, Taichung City 404, Taiwan

**Keywords:** antiplatelet, glutaminolysis, obesity, platelet

## Abstract

Obesity is associated with a wide range of chronic diseases, including cancer. It has been noted that the integration of metabolic mechanisms in obese patients may predispose them to suffer from cancer incidence and its progression. Thus, a better understanding of metabolic alterations in obesity, along with the development of feasible therapeutic approaches for intervention, are theoretically relevant to the prevention and treatment of cancer malignancy. Using a syngeneic tumor model involving Lewis Lung Carcinoma (LLC) cells and C57BL/6 mice fed with a high fat diet, obesity was found to be associated with dysregulated glucose and glutamine metabolism, inflammation, along with platelet activation and the promotion of tumor growth. Tumor-bearing lowered glucose levels while moderately increasing inflammation, platelet activation, and glutamine levels. The antiplatelet drug aspirin, mitigated tumor growth in obese mice, paralleled by a decrease in systemic glucose, insulin, inflammation, platelet activation, glutamine and tumor expression of cell proliferation, aerobic glycolysis, glutaminolysis, platelets, and leukocyte molecules. The anti- and pro-cell proliferation, aerobic glycolysis, and glutaminolysis effects of aspirin and glutamine were further demonstrated in a LLC cell study. Although there remains limitations to our experiments, glucose and glutamine metabolism are proposed targets for the anticancer effects of aspirin.

## 1. Introduction

Obesity is a global concern, which brings upon major health problems. Beyond its immediate and obvious health significance, obesity is also associated with an increased risk and poorer outcome for many chronic diseases, including cancer [1]. Hyperglycemia, hyperinsulinemia, and chronic, low-grade inflammation are hallmarks of obesity. It has been postulated that hyperglycemia, hyperinsulinemia, adipokines, and cytokines in obese subjects provide abundant nutrients and growth factors for cancer cells, resulting in the establishment of an appropriate niche for tumor progression and malignancy [1,2,3]. Obesity may integrate several metabolic mechanisms to predispose suffering from cancer incidence and progression. Thus, a better understanding of metabolic alterations surrounding obesity, along with the development of feasible therapeutic approaches for intervention are theoretically relevant to the treatment and prevention of cancer malignancy.

Hyperglycemia and dyslipidemia are two metabolic alterations of obesity, which suggest therapeutic options for obesity comorbidities with glucose or lipid targeting. Evidence indicates that statins, metformin, and sodium-glucose cotransporter-2 inhibitors slow tumor growth and reverse the obesity-driven aggressiveness of cancer [1,2,4,5]. Additionally, platelet numbers, platelet activation, and platelet-derived microparticles are positively associated with obese individuals [6,7,8]. Pre-diagnostic high platelet counts indicate a poor prognosis for cancer patients, and play a role in the responsiveness to targeted therapy, radiotherapy, and chemotherapy [9,10,11,12,13,14]. Platelets and their released mediators interact with tumors to coordinate a tumor-prone vasculature and microenvironment via mutual crosstalk. Specifically, Vascular Endothelial Growth Factor (VEGF), Transforming Growth Factor-β1 (TGF-β1), High Mobility Group Box-1 (HMGB1), glutamine, and glutaminase are reported to be mediators of the platelet-driven malignant progression of cancer [15,16,17,18]. Since platelets not only contribute to hemostasis but also to cancer pathogenesis, antiplatelet drugs represent alternative candidates for cancer treatment.

Aspirin is a widely used antiplatelet drug. Beyond its cardiovascular and cerebrovascular effects, epidemiological, clinical, and experimental evidence has shown that aspirin plays certain roles with regards to anticancer. Aspirin acts directly on platelets, leading to the inhibition of platelet-driven cancer malignancy and the formation of metastatic intravascular niches [19,20,21]. The platelet- and cyclooxygenase-independent anticancer effects of aspirin include acting on Hypoxia-Inducible Factor-1α (HIF-1α), exosome, heparanase, NF-κB, Epithelial-Mesenchymal Transition (EMT), Pyruvate Kinase M2 (PKM2), p21, pentose phosphate pathway, and inflammation [13,22,23,24,25,26,27,28]. Despite its advanced achievements, the anticancer effects of aspirin are still not completely understood.

Other than the conventional nude mice xenograft tumor model, the syngeneic tumor model of Lewis Lung Carcinoma (LLC) cells and C57BL/6 mice has been previously established for the study of cancer-associated thrombosis [29]. Additionally, C57BL/6 mice are also commonly used in the study of diet-induced obesity [30]. To extend the scope of aspirin studies, this study was conducted to identify any metabolic alterations in obese mice and to also investigate the anticancer effects of aspirin on obesity-driven cancer malignancy, by taking advantage of the syngeneic tumor model.

## 2. Materials and Methods

### 2.1. Cell Culture

Murine Lewis Lung Carcinoma (ATCC CRL-1642^TM^) cells were maintained in a Dulbecco’s Modified Eagle Medium (DMEM) with 4 mM L-glutamine containing 10% Fetal Bovine Serum (FBS). To conduct experiments of aspirin effects, cells were placed in a DMEM with 4 mM L-glutamine containing 5% FBS. For the study of glutamine effects, cells were maintained in DMEM without L-glutamine containing 5% FBS and were exogenously supplemented with various concentrations of L-glutamine. Since LLC cells displayed remarkable cytotoxicity in glutamine-free DMEM, the minimal concentration of L-glutamine in the relevant studies was 1 mM.

### 2.2. Cell Viability Assay

LLC cells were seeded onto a 96-well plate. An assay kit (CellTiter 96^®^ AQ_ueous_ Non-Radioactive Cell Proliferation Assay Kit, Promega, Madison, WI, USA) was utilized to measure cell viability in accordance with the manufacturer’s instructions.

### 2.3. Caspase 3 Activity Assay

A Caspase Fluorometric Assay Kit (BioVision, Mountain View, CA, USA) was utilized to measure caspase 3 activity according to the instructions. The intensity of the fluorescence signals measured with a fluorometer (E_x_ 380 nm and E_m_ 460 nm) was normalized by protein contents and the relative activity was expressed.

### 2.4. Syngeneic Tumor Model Study

The protocols of animal study had been reviewed and approved by the Animal Experimental Committee of Taichung Veterans General Hospital (ethical code number: La-1081613, date: 10 January 2019). Male C57BL/6 mice (8 weeks old) were separately fed with a normal diet (ND, 10% energy from fat, 58Y2, TestDiet, St. Louis, MO, USA) or a high fat diet (HFD, 60% energy from fat, 58Y1, TestDiet, St. Louis, MO, USA) for 10 weeks. Then, LLC cells (1 × 10^5^ cells in 100 µL of serum-free DMEM) or equal volumes of normal saline were inoculated subcutaneously into the right flanks of the mice. To investigate the effects of obesity on tumor growth, mice were allocated into four groups containing ND/Saline, ND/LLC, HFD/Saline, and HFD/LLC (n = 5 per group). After LLC cells inoculation, mice were continuously fed with corresponding ND or HFD for an additional two weeks. For the study of aspirin effects, mice were divided to three groups including ND/LLC/Saline, HFD/LLC/Saline, and HFD/LLC/Aspirin (n = 6 per group). Three days after LLC cells inoculation, mice began to receive daily doses of aspirin (20 mg/kg, ip) or saline administration, and continued to be fed with corresponding ND or HFD for 18 days. At the end of the two experiments, the mice were sacrificed and their tumors were resected for measurement and analyses. Tumor volume was calculated according to the following formula: V = (L × W^2^)/2 (L = length; W = width) [31].

### 2.5. Colony Formation Assay

LLC cells (500 cells per well) were plated onto a 6-well plate in a DMEM supplemented with 5% FBS. One day after seeding, the cells were treated with various concentrations of indicated agents for 6 days. After fixation, the cell colonies were visualized through the use of staining with crystal violet.

### 2.6. Blood Sample Collection and Analyses

At the end of studies, mice were deprived of any food for 8 hours prior to sacrifice. After anesthesia with isoflurane, blood samples were withdrawn from the left femoral artery. The numbers of White Blood Cells (WBC), neutrophils, lymphocytes, and platelets were measured by complete blood count. The fasting glucose level was then measured using a hand-held Accucheck glucometer (Roche Diagnostics, Indianapolis, IN, USA). The plasma levels of insulin (Shibayagi, Gunma, Japan), leptin (R&D Systems, Minneapolis, MN, USA), soluble P-selectin (R&D Systems, Minneapolis, MN, USA), TGF-β1 (R&D Systems, Minneapolis, MN, USA), and glutamine (Abcam, Cambridge, MA, USA) were determined using enzyme immunosorbent assay (ELISA) kits, following the procedures provided by the respective manufacturers. Insulin resistance was evaluated according to the Homeostasis Model Assessment (HOMA) [32]. The HOMA Insulin Resistance (HOMA-IR) index was calculated as [fasting insulin (μU/mL) × fasting glucose (mmol/L)]/22.5.

### 2.7. Western Blot

Cells and resected tumor tissues were subjected to protein extraction using tissue protein reagents (T-PER, Pierce Biotechnology, Rockford, IL, USA). Proteins were separated by SDS-PAGE and then transferred to PVDF membranes. The membranes were incubated with the following antibodies: cyclin D1, β-catenin, Src, phospho-Src, Akt, phospho-Akt, Smad2/3, phospho-Smad2/3, Thromboxane A2 Receptor (TXA2R) (Santa Cruz Biotechnology, Santa Cruz, CA, USA), Glucose Transporter-1 (GLUT1), PKM2, SLC1A5, SLC7A5, glutaminase, CD41, CD62P, CD45, SDF-1α, CXCR4 (Abcam, Cambridge, MA, USA), and Glyceraldehyde-3-phosphate Dehydrogenase (GAPDH) (R&D Systems, Minneapolis, MN, USA). The reacted proteins were further determined with horseradish peroxidase-labeled IgG, visualized using Enhanced Chemiluminescence (ECL) Western blotting reagents, and quantified by the optical densitometry (Image Master ID, Pharmacia Biotech, Upsalla, Sweden) of developed autoradiographs.

### 2.8. Statistical Analysis

Data are expressed as means ± standard deviations. Statistical comparisons were analyzed using a one-way analysis of variance, followed by the Tukey’s or Dunnett’s test. A p value less than 0.05 was considered statistically significant.

## 3. Results

### 3.1. Tumor Improved Glucose Metabolism in Obese Mice

**Figure 1:**
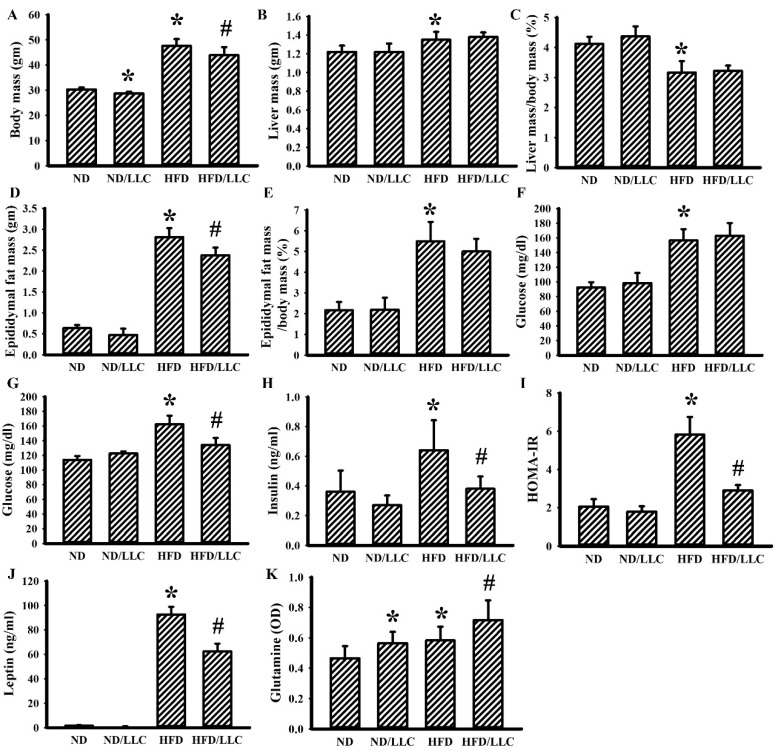
Tumor growth improved glucose metabolism in obese mice. LLC cells (LLC) or saline vehicle were implanted into the lean (normal diet, ND) and obese (high fat diet, HFD) mice, and grew for 2 weeks. The body mass (**A**), liver mass (**B**), relative percentage of liver mass (**C**), epididymal fat mass (**D**), and relative percentage of epididymal fat mass (**E**) were weighed and analyzed. Fasting glucose levels were measured at times of pre-implanted (**F**) and post-implanted (**G**). Fasting insulin level (**H**), Homeostasis Model Assessment-Insulin Resistance (HOMA-IR) (**I**), leptin level (**J**), and glutamine level (**K**) were determined. * *p* < 0.05 vs. ND and # *p* < 0.05 vs. HFD (n = 5 per group).

**Figure 2:**
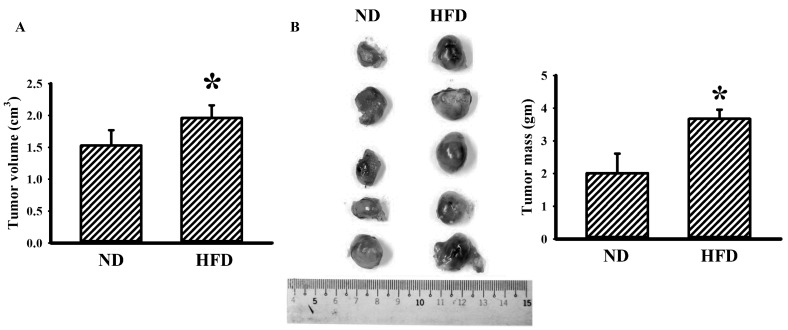
Obesity promoted tumor growth. LLC cells (LLC) were implanted into the lean (ND) and obese (HFD) mice and grew for 2 weeks. The tumor volume (**A**) and tumor mass (**B**) were weighed and analyzed. The resected tumors are shown (**B**). * *p* < 0.05 vs. ND (n = 5 per group).

When compared with ND-fed mice, HFD-fed mice developed obesity (Figure 1A–E), hyperglycemia (Figure 1F,G), hyperinsulinemia (Figure 1H), insulin resistance (Figure 1I), hyperleptinemia (Figure 1J) and promoted tumor growth (Figure 2). Tumor-bearing ND-fed mice had little effect on the metabolic parameters, except for body mass, while tumor-bearing HFD-fed mice showed a decrease in metabolic parameters other than liver mass, relative percentage of liver mass, and relative percentage of epididymal fat mass (Figure 1). Additionally, tumor-bearing ND-fed mice and HFD-fed mice showed increased plasma glutamine, with the level further elevated in tumor-bearing HFD-fed mice (Figure 1K). This implies that tumor-bearing in HFD-fed mice tends to decrease body mass, epididymal fat mass, plasma glucose, plasma insulin, plasma leptin, and insulin resistance, while elevating plasma glutamine.

### 3.2. Tumor Elevated Circulating Leukocytes in Obese Mice

**Figure 3:**
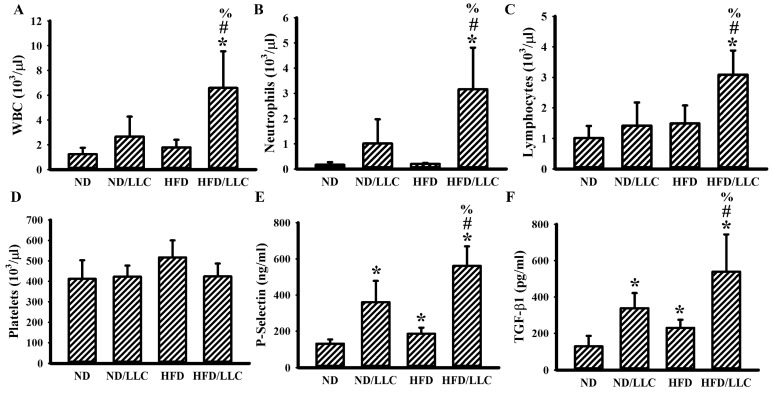
Tumor growth augmented inflammation in obese mice. LLC cells (LLC) or saline vehicle were implanted into the lean (ND) and obese (HFD) mice and grew for 2 weeks. The total white blood cells (WBC) (**A**), neutrophils (**B**), lymphocytes (**C**), platelets (**D**), soluble P-selectin (**E**), and Transforming Growth Factor-β1 (TGF-β1) (**F**) in blood samples were determined. * *p* < 0.05 vs. ND, % *p* < 0.05 vs. ND/LLC, and # *p* < 0.05 vs. HFD (n = 5 per group).

In syngeneic tumor mice, tumor and HFD feeding alone seemed to have little effect on the numbers of circulating total WBC (Figure 3A), neutrophils (Figure 3B), and lymphocytes (Figure 3C). However, tumor-bearing HFD-fed mice showed an increased number of WBC (Figure 3A), neutrophils (Figure 3B), and lymphocytes (Figure 3C). Although the numbers of platelets were constant amongst groups (Figure 3D), the concentrations of soluble P-selectin (Figure 3E) and TGF-β1 (Figure 3F) were elevated in three groups when compared with ND-fed mice, with the most being tumor-bearing HFD-fed mice. Despite having little effect on cell numbers, the findings indicate that both tumor-bearing and HFD feeding tend to activate platelets and cause TGF-β1 production, particularly in the group of tumor-bearing and HFD feeding mice.

### 3.3. Aspirin Improved Metabolic and Inflammatory Alterations in Tumor-Bearing HFD-Fed Mice

**Figure 4:**
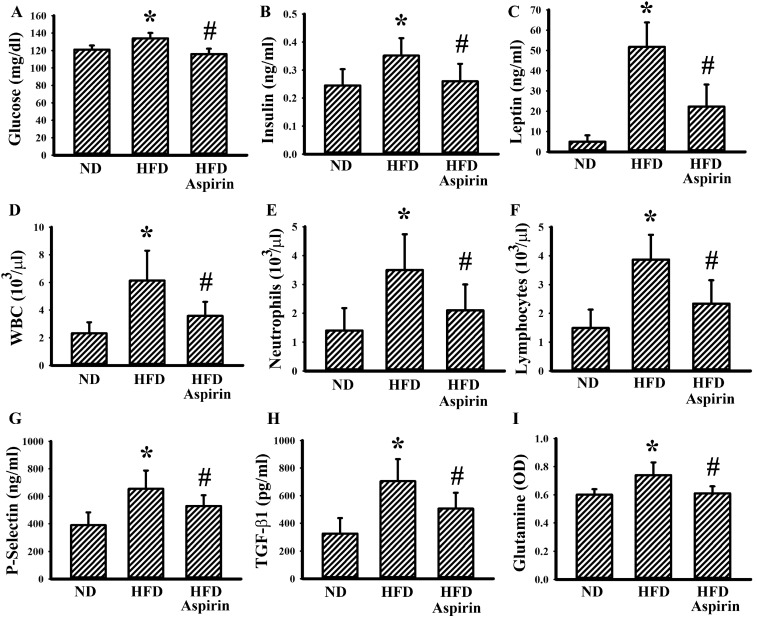
Aspirin improved metabolic alterations in obese mice. LLC cells (LLC) were implanted into the lean (ND) and obese (HFD) mice and grew for 3 weeks. Three days after implantation, aspirin (20 mg/kg) was administrated daily up until the end of the experiment. The fasting glucose (**A**), insulin (**B**), leptin (**C**), WBC (**D**), neutrophils (**E**), lymphocytes (**F**), soluble P-selectin (**G**), TGF-β1 (**H**), and glutamine (**I**) in blood samples were determined. * *p* < 0.05 vs. ND and # *p* < 0.05 vs. HFD (n = 6 per group).

**Figure 5:**
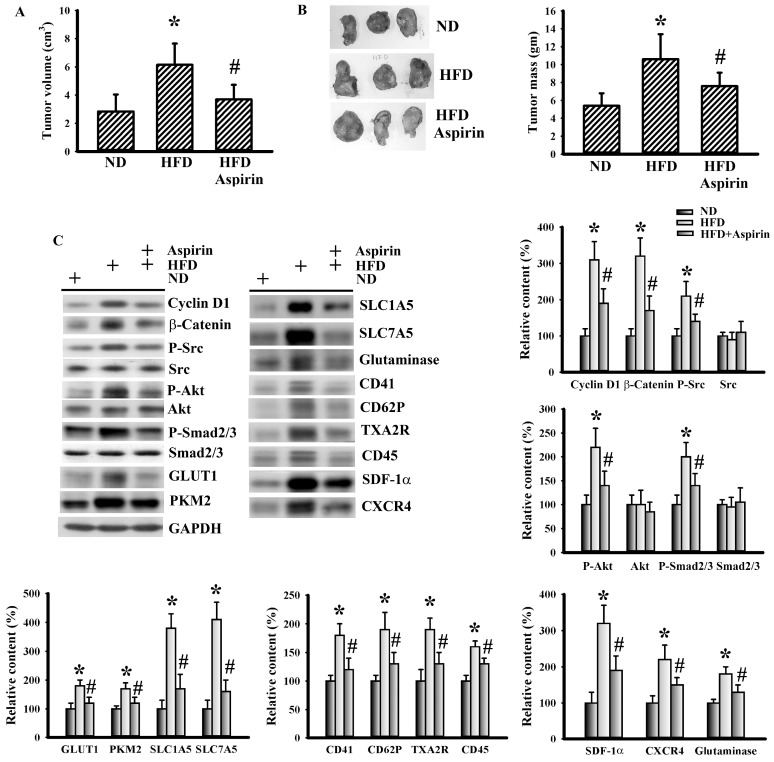
Aspirin mitigated tumor growth in obese mice. LLC cells (LLC) were implanted into the lean (ND) and obese (HFD) mice and grew for 3 weeks. Three days after implantation, aspirin (20 mg/kg) was administrated daily up until the end of the experiment. The tumor volume (**A**) and tumor mass (**B**) were weighed and analyzed. The representative resected tumors are shown (**B**). Proteins were extracted from the resected tumor tissues and subjected to Western blot with indicated antibodies. Representative blot is shown (**C**). Quantitative data are depicted and the content in ND was defined as 100%. * *p* < 0.05 vs. ND and # *p* < 0.05 vs. HFD (n = 6 per group).

To understand the effects of aspirin, changes of metabolic alterations were first examined. Aspirin mitigated the increments of glucose, insulin, leptin, WBC, neutrophils, lymphocytes, glutamine, soluble P-selectin, and TGF-β1 in tumor-bearing HFD-fed mice (Figure 4). In a parallel study, the growth of tumor mass was slowed by aspirin administration (Figure 5A,B). This implies that the changes may be secondary to the anticancer effects of aspirin, or that they play substantial roles regarding the anticancer effects of aspirin.

### 3.4. Aspirin Mitigated Cellular Metabolic Capacity and Cell Growth in Tumor-Bearing HFD-Fed Mice

In order to correlate with the systemic changes of metabolic alterations, inflammatory responses, and platelet activation, the relevant expression of corresponding molecules in tumor tissues was examined. First, we observed that the inhibition of tumor growth due to aspirin was accompanied by alterations in several cell proliferation-associated transcription factors and signaling molecules. Tumors of HFD-fed mice increased their expression of proliferation-promoting cyclin D1, β-catenin, Src phosphorylation, Akt phosphorylation, and Smad phosphorylation when compared with tumors in ND-fed mice, with the changes in tumor-bearing HFD-fed mice being reversed by aspirin (Figure 5C). The expressions of aerobic glycolysis-associated GLUT1 and PKM2 [29], and glutaminolysis-associated SLC1A5, SLC7A5, and glutaminase [18] were all elevated in tumors of HFD-fed mice, which was mitigated by aspirin (Figure 5C). Additionally, platelet-associated CD41, CD62P, and TXA2R [33], leukocyte-associated CD45 and pleiotropic SDF-1α, along with CXCR4, were all elevated in tumors of HFD-fed mice and alleviated by aspirin (Figure 5C). This would imply that HFD-promoted tumor growth is accompanied by increased cell proliferation, aerobic glycolysis, glutaminolysis, and platelet and leukocyte tumor infiltration, with the biochemical events being targeted by aspirin.

### 3.5. Aspirin Mitigated LLC Cell Proliferation and Metabolism

**Figure 6:**
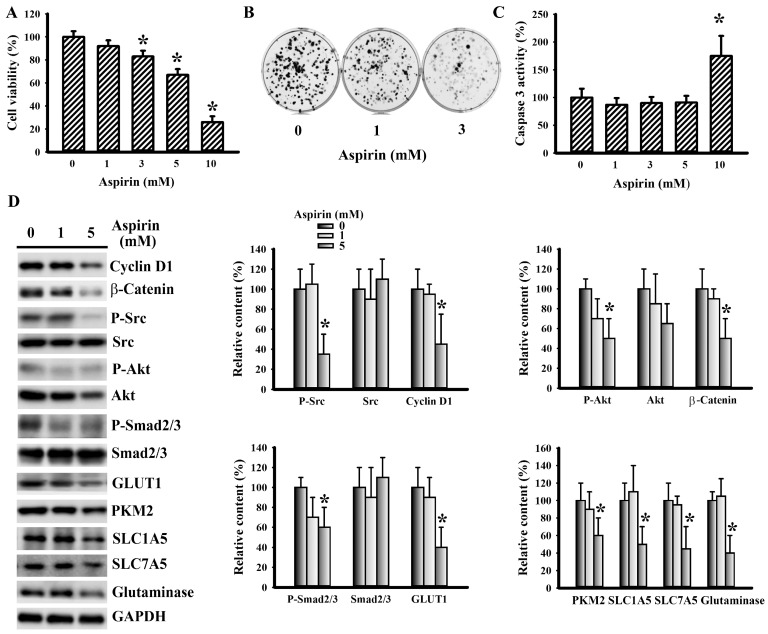
Aspirin mitigated LLC cell proliferation. (**A**) LLC cells were treated with various concentrations of aspirin for 24 h. Cell viability was evaluated by MTS reduction assay. (**B**) LLC cells were treated with various concentrations of aspirin for 6 days. Cell colonies were fixed and stained with crystal violet. (**C**) LLC cells were treated with various concentrations of aspirin for 16 h. Proteins were extracted and subjected to enzymatic assay of caspase 3 activity. (**D**) LLC cells were treated with various concentrations of aspirin for 16 h. Proteins were extracted and subjected to Western blot with indicated antibodies. Representative blot is shown. Quantitative data are depicted and the content in untreated control was defined as 100%. * *p* < 0.05 vs. untreated control (n = 3).

To further explore its anticancer effects, the responses of LLC cells to aspirin were examined. Aspirin caused a reduction of cell viability (Figure 6A) and long-term clonogenesis (Figure 6B), while having little effect on caspase 3 activity, except in the highest concentration (10 mM) (Figure 6C). To establish the potential involvement of the aforementioned molecules in aspirin-induced LLC cell growth inhibition, their expressions were measured. A decreased expression of cyclin D1, β-catenin, Src phosphorylation, Akt phosphorylation, Smad2/3 phosphorylation, GLUT1, PKM2, SLC1A5, SLC7A5, and glutaminase was noted in aspirin-treated cells (Figure 6D). These findings indicate that aspirin has a negative effect on cell proliferation, as well as glucose and glutamine utilization.

### 3.6. Glutamine Promoted LLC Cell Proliferation and Metabolism

**Figure 7:**
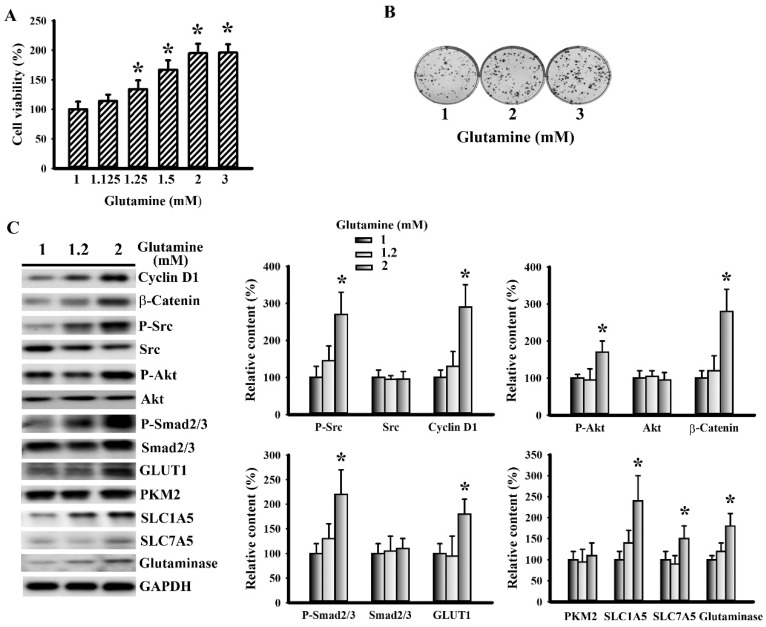
Glutamine promoted LLC cell proliferation. (**A**) LLC cells were treated with various concentrations of glutamine for 24 h. Cell viability was evaluated by MTS reduction assay. (**B**) LLC cells were treated with various concentrations of glutamine for 6 days. Cell colonies were fixed and stained with crystal violet. (**C**) LLC cells were treated with various concentrations of glutamine for 16 h. Proteins were extracted and subjected to Western blot with indicated antibodies. Representative blot is shown. Quantitative data are depicted and the content in untreated control was defined as 100%. * *p* < 0.05 vs. untreated control (n = 3).

The aforementioned findings imply that glutaminolysis may be a target of the anticancer effect of aspirin; therefore, the direct actions of glutamine on LLC cells were examined. Unlike aspirin, exogenous glutamine increased cell viability (Figure 7A) and long-term clonogenesis (Figure 7B). The growth-promoting effect of glutamine was associated with upregulated cyclin D1, β-catenin, Src phosphorylation, Akt phosphorylation, Smad2/3 phosphorylation, GLUT1, SLC1A5, SLC7A5, and glutaminase expression (Figure 7C). This would imply that glutamine has a positive effect on cell proliferation, as well as glucose and glutamine utilization.

## 4. Discussion

Obesity and cancer are two metabolic diseases. Epidemiological, clinical, and experimental studies have shown that long-term use of aspirin is associated with a low incidence of cancer, low risk of malignant transform and metastasis, and low cancer mortality [34,35]. Using a syngeneic tumor model with LLC cells and C57BL/6 mice, we herein demonstrated that obese mice showed signs of hyperglycemia, hyperinsulinemia, insulin resistance, hyperleptinemia, low-grade systemic inflammation, platelet activation, glutamine elevation, and augmented tumor growth. Tumor growth moderately improved the metabolic alterations of glucose while augmenting systemic inflammation, platelet activation, and glutamine elevation in obese mice. Aspirin mitigated tumor growth in obese mice with lowered glucose, insulin, leptin, leukocyte number, glutamine, TGF-β1, and platelet activation. Parameters of cell proliferation, aerobic glycolysis, glutaminolysis, along with platelet and leukocyte tumor infiltration were elevated in tumors of obese mice and reversed by aspirin. Rather that causing apoptotic cell death, aspirin slowed cell proliferation as well as glucose and glutamine utilization in vitro. Oppositely, glutamine increased cell proliferation as well as glucose and glutamine utilization. Therefore, glucose and glutamine metabolism appear to have a role in obesity-driven cancer malignancy, and represent targets of aspirin regarding anticancer.

Obesity is associated with an increased risk in a range of various types of cancers. Specifically, hyperglycemia, hyperinsulinemia, adipokines, and cytokines coordinate the integration of nutritional, mitogenic, vascular, and inflammatory cues in favoring tumor progression and malignancy [1,2,3]. Our findings of hyperglycemia, hyperinsulinemia, hyperleptinemia, the circulating number of WBC, neutrophils, and lymphocytes, plasma TGF-β1, along with tumor CD45, SDF-1α, and CXCR4 in obese mice, were consistent with the reported studies. Intriguingly, the hyperglycemia, hyperinsulinemia, insulin resistance, and hyperleptinemia in obese mice were improved by tumor-bearing. In obesity-associated dysfunction during glucose metabolism, the impairment of insulin action on the peripheral organs is typically of importance, particularly at the skeletal muscles [36]. In our study, the insulin signaling in the gastrocnemius muscles of obese mice was impaired (data not shown). However, tumors of obese mice expressed increased Akt phosphorylation, GLUT1, and PKM2. Therefore, the upregulated Akt signaling, glucose uptake, and metabolism in tumor tissues may be the causes surrounding the improvement in glucose utilization. It should be noted that this improvement may also be secondary to the weight loss since tumor-bearing caused a reduction in both body mass and epididymal fat mass.

Tumor cells adopt a unique metabolic strategy by coupling aerobic glycolysis and glutaminolysis [18,37,38]. GLUT1 and PKM2 play a key role in glucose uptake and aerobic glycolysis [26]. Whereas SLC1A5 and SLC7A5 transport extracellular glutamine into the cells, and glutaminase metabolizes glutamine to glutamate for the completion of the citric acid cycle, GSH synthesis, and pentose phosphate pathway [18,38]. An additional study has shown that glutamine is beneficial to hepatic glucose homeostasis in obese mice [39]. Here, we found that tumor-bearing caused an increase in the plasma glutamine level and tumor expression of SLC1A5, SLC7A5, and that glutaminase was further elevated in obese mice. These findings have inspired us to speculate that both the glucose and glutamine supplies may be a link between obesity and cancer malignancy. In vitro, glutamine directly promoted LLC cell proliferation and survival, with an increase in Src, Akt, and TGF-β1/Smad signaling, aerobic glycolysis, and glutaminolysis. Since both glucose and glutamine possess a diversity of biological effects, our hypothesis warrants a deeper investigation.

Other important findings taken from this current study were the activation and tumor infiltration of platelets in tumor-bearing mice, particularly obese mice. There have already been many studies, which have shown a pro-malignant role for platelets. Platelet aggregates, released factors, and tumor infiltration have been implicated throughout the different steps of carcinogenesis; including proliferation, escape of immune surveillance, extravasation, and metastasis [9,10,11,12,13,14,15,16,17,18]. Platelets express CD41 and CD62P and are the major source of circulating soluble P-selectin. For the activation, TXA2R is a potent stimulator of platelet aggregation [33,40]. Apart from platelets- and activated platelets-associated soluble P-selectin, CD41, CD62P, and TXA2R, glutamine, glutaminase, and TGF-β1 are also bioactive molecules released by platelets [15,16,17,18,33]. TGF-β1 is a factor in inducing glutaminase expression, with its expression being under the control of the SDF-1α/CXCR4 axis [41,42,43]. Although their coexistence was discovered in the current study, the exact biological implications and mutual interactions were not addressed.

Aspirin is a more than century old medication and a commonly prescribed drug. Its chemopreventive effects are increasingly valued. Platelet- and cycloxygenase-dependent and independent mechanisms have been proposed regarding the anticancer effects of aspirin in cell models and rodent models of both nude mice and lean mice [13,19,20,21,22,23,24,25,26,27,28]. Aspirin has been found to inhibit tumor growth in obese mice through lowered plasma glucose, insulin, leptin, WBC, neutrophils, lymphocytes, soluble P-selectin, TGF-β1, and glutamine. Biochemically, tumor cell proliferation, aerobic glycolysis, glutaminolysis, as well as platelet and leukocyte infiltration were all alleviated through the use of aspirin. Additionally, LLC cell proliferation, aerobic glycolysis, and glutaminolysis were directly mitigated by aspirin, in vitro. Although TGF-β1, PKM2, β-catenin, and NF-κB have been implicated in the anticancer effects of aspirin [24,25,26,28], its effect on glutaminolysis remains largely unclear. Current in vivo and in vitro findings have suggested its potential effect on glutaminolysis. There is evidence showing a beneficial effect of aspirin against insulin resistance [44]. Thus, the metabolic effects on glucose and glutamine may represent alterative targets for aspirin with regards to anticancer action. It should be noted that the concentrations of aspirin used in current cell studies (1–5 mM) are relatively higher than the therapeutic aspirin concentrations (~0.5 mM). The translation of current findings to clinical implication warrants a deeper investigation.

## 5. Conclusions

In conclusion, using a syngeneic tumor model with LLC cells and C57BL/6 mice, obesity was associated with dysregulated glucose and glutamine metabolism, inflammation, and platelet activation, as well as the promotion of tumor growth. Tumor-bearing lowered glucose levels, while it moderately increased inflammation, platelet activation, and glutamine levels. Aspirin alleviated tumor growth in obese mice, paralleled by a decrease in systemic glucose, insulin, inflammation, platelet activation, and glutamine, along with the tumor expression of cell proliferation, aerobic glycolysis, glutaminolysis, platelets, and leukocyte molecules. The anti- and pro-cell proliferation, aerobic glycolysis, and glutaminolysis effects of aspirin and glutamine were further demonstrated in an LLC cell study. The anticancer effects of the antiplatelet drug aspirin are multifactorial. Although limitations still remain regarding our experiments, glucose and glutamine metabolism are proposed targets for the anticancer effects of aspirin. Unfortunately, the exact cell types and sources of the molecules released were not analyzed during the current study.
